# Ortner’s syndrome after cryoballoon ablation

**DOI:** 10.1007/s10840-018-0466-4

**Published:** 2018-10-23

**Authors:** Grzegorz Karkowski, Stanislas Kielczewski, Jacek Lelakowski, Marcin Kuniewicz

**Affiliations:** 1 Department of Electrocardiology, Institute of Cardiology, John Paul II Hospital, Jagiellonian University Medical College, Krakow, Poland; 2 Department of Anatomy, Jagiellonian University Medical College, Krakow, Poland

We present the case of a 54-year-old patient with hypertension, obesity (BMI, 31), paroxysmal atrial fibrillation EHRA class III B, a CHADSVASc score of 4, a HAS-BLED score of 2, a history of TIA, and an ineffective epicardial ablation for atrial fibrillation using the COBRA Fusion system with simultaneous ligation of the left atrial appendage 8 months prior.

For the repeat ablation procedure, we selected cryoballoon ablation (Arctic Front Advance 28 mm). Using the Achieve diagnostic catheter, we identified potentials in the left superior pulmonary vein (LSPV) with no exit block. After contrast injection, ablation of the LSPV was performed at − 58 °C until disappearance of pulmonary vein potentials in 30-s applications (cumulative time 150 s). The final result was complete pulmonary venous isolation together with exit and entrance block. During the procedure, the patient was sedated and anesthetized with midazolam and fentanyl. Hoarseness was observed in the early postoperative period. A laryngological examination revealed a left unilateral vocal fold paralysis. A 14-day course of oral prednisone did not resolve the hoarseness.

**Fig. 1:**
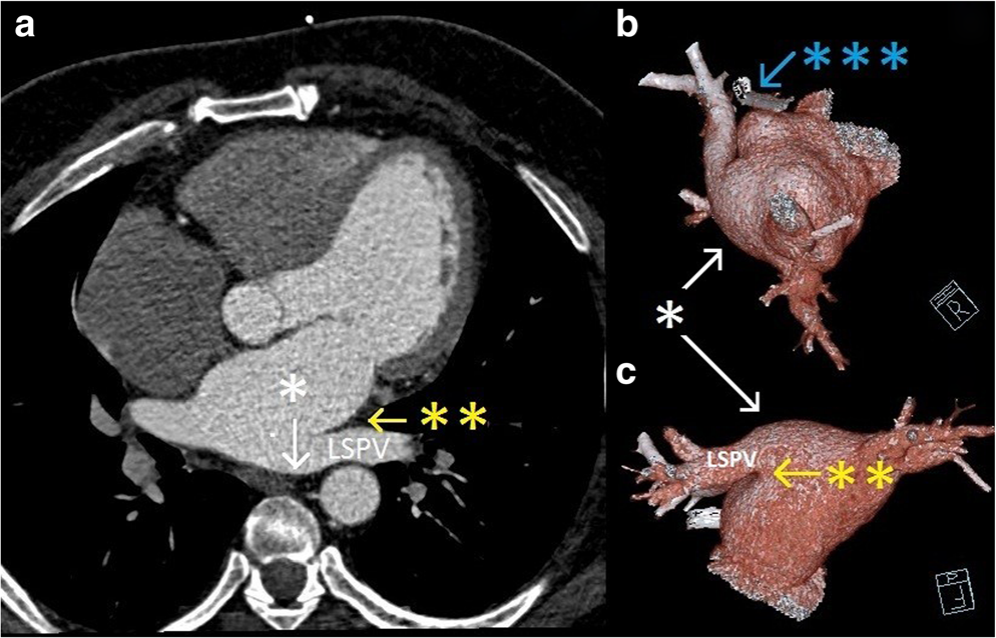
Angio-CT scan of the left atrium (A) and 3D reconstructions (B and C). Single asterisk denotes a bulging roof of the left atrium (white); double asterisks, a grove after epicardial box ablation (yellow); and triple asterisks, LAA AtriClip (blue)

In the post-cryoballoon ablation heart CT with contrast, a scar was noted along the epicardial ablation with a change in the architecture of the roof of the left atrium (Fig. 1). After ineffective steroid therapy, we initiated a 10-week course of intramuscularly administered Milgamma (vitamin B complex, cyanocobalamin 1 mg, pyridoxine 100 mg, and thiamine 100 mg). After 7 days of this therapy, the hoarseness resolved. At follow-up 4 months after the procedure, neither the atrial fibrillation nor the hoarseness has recurred.

Ablation is an important and constantly developing method of treatment of atrial fibrillation (AF) [1]. Ablations using the COBRA Fusion system may be incomplete in the anterior-superior part of the LSPV due to fat thickness along the roofline [2]. LRLN injury was described once during RF ablation over the roof line in close relation to LSPV [3], but never before during cryoballoon ablation. Authors suggested deformation of the atrial wall by a stiff electrode could increase the likelihood of contact with the LRLN [3]. In our case, there was a post-lesion box change in the atrial architecture on the roof after the first ablation which could cause the same effect. This meant the LRLN was within a range of cryoenergy (− 58 °C) that was delivered in the LSPV. These circumstances support the diagnosis of iatrogenic Ortner’s syndrome, which was first described in 1897.

Recovery from laryngeal nerve palsy after thermal damage might be spontaneous or with a late response to prednisone therapy, but the effectiveness in recovery after administration of vitamin B combination complex was interesting.
